# Archaeal viruses at the cell envelope: entry and egress

**DOI:** 10.3389/fmicb.2015.00552

**Published:** 2015-06-05

**Authors:** Emmanuelle R. J. Quemin, Tessa E. F. Quax

**Affiliations:** ^1^Department of Microbiology, Institut Pasteur, Paris, France; ^2^Molecular Biology of Archaea, Institute for Biology II - Microbiology, University of Freiburg, Freiburg, Germany

**Keywords:** archaea, archaeal virus, bacterial virus, virion entry, virion egress, archaeal membrane, pili, lysis

## Abstract

The cell envelope represents the main line of host defense that viruses encounter on their way from one cell to another. The cytoplasmic membrane in general is a physical barrier that needs to be crossed both upon viral entry and exit. Therefore, viruses from the three domains of life employ a wide range of strategies for perforation of the cell membrane, each adapted to the cell surface environment of their host. Here, we review recent insights on entry and egress mechanisms of viruses infecting archaea. Due to the unique nature of the archaeal cell envelope, these particular viruses exhibit novel and unexpected mechanisms to traverse the cellular membrane.

## Introduction

Members of the three domains of life, Archaea, Bacteria and Eukarya, are all subject to viral infections. Viruses have been isolated from various environments, where they are often abundant, outnumbering prokaryotic cells by a factor of 10 (Bergh et al., 1989; Borsheim et al., 1990; Suttle, 2007). Viruses infecting archaea tend to display high morphological and genetic diversity compared to viruses of bacteria and eukaryotes (Pina et al., 2011). Several archaeal viral families have members, which display unique shapes that are not found amongst other viruses, such as a bottle, droplet or spiral (Prangishvili, 2013).

The cell envelope represents a major barrier for all viruses. In fact, the cell membrane has to be traversed twice by viruses to establish successful infection, first upon entry and secondly during exit. In order to cross the cell envelope, viruses have developed various strategies, each adapted to the membrane environment of their host.

The combination of high-throughput approaches with more classical techniques has shed light on the process of viral entry and release in some archaeal virus-host model systems. However, the detailed molecular mechanisms underlying the various stages of the viral life cycle remain poorly understood in archaea in general (Quemin et al., 2014). Recently, a few studies have focused on the adsorption at the surface of the archaeal host cell before viral entry and release of viral particles at the end of the infection cycle (Bize et al., 2009; Brumfield et al., 2009; Ceballos et al., 2012; Quemin et al., 2013; Deng et al., 2014). This has delivered the very first insights into the fashion in which viruses interact with the archaeal membrane.

The cell surface of archaea is fundamentally different from bacteria (Albers and Meyer, 2011). Archaeal membranes have an alternative lipid composition and generally lack a cell wall of peptidoglycan. In addition, the motility structures present at the surface of archaea are constructed from different building blocks than their bacterial counterparts (Pohlschroder et al., 2011). Gram positive bacteria contain a lipid bilayer covered by a thick peptidoglycan cell wall and gram negative cells are surrounded by two membranes with a thinner peptidoglycan in the periplasmic space in between. While bacteria typically contain a cell wall polymer of peptidoglycan (Typas et al., 2012), peptidoglycan cell walls are absent from archaea. Instead, most archaea are surrounded by a thin proteinaceous surface layer (S-layer) that consists of glycosylated proteins, which are anchored in the cell membrane. In contrast to the peptidoglycan, which has a molecular composition that can be very similar from one species to another, S-layer proteins show a great diversity (Fagan and Fairweather, 2014). Hence, archaea exhibit specific features, in particular at the cell surface, which are not shared with bacteria and influence the mechanisms at play in the course of infection.

The first studies on archaeal viral entry and egress have shown that some archaeal viruses employ entry strategies that superficially resemble those of bacterial viruses (Quemin et al., 2013; Deng et al., 2014), while others utilize surprisingly novel exit mechanisms (Brumfield et al., 2009; Quax et al., 2011). Here we will give an overview of the first studies reporting viral interaction with the archaeal cell envelope, focusing on hyperthermophilic crenarchaeal viruses. Furthermore, current research permits comparison with corresponding mechanisms taking place during the viral cycle of bacterial viruses. We will discuss how features of cell surfaces compel viruses to employ specific strategies for entry and egress.

## Viral Entry

A virus is able to infect only a few strains or species. Such specificity in interaction of viruses with their host is determined by the characteristics of entry, which in turn rely on the nature and structural peculiarities of the cell envelope. Adsorption as the first key step of the viral cycle is one of the most restrictive in terms of host range, depending on the accessibility and number of receptors present at the cell surface (Poranen et al., 2002). Structural proteins are found within the viral particle in metastable conformation and it is the interaction with the host cell, which leads to a more stable, lower-energy conformation of these proteins (Dimitrov, 2004). Indeed, virus entry and genome uncoating are energy-dependent processes and irreversible conformational change of the capsid proteins (CP) during adsorption triggers the release of the genome from the extracellular virions (Molineux and Panja, 2013). As a general rule, entry can be subdivided in two steps. For the well-studied viruses infecting bacteria, the first contact with the host is reversible and then, viruses attach irreversibly to a specific, saturable cell envelope receptor. Primary and secondary adsorptions can take place with the same receptor or, more frequently involve different players. Common cellular determinants in bacteria are peptidoglycan, lipopolysaccharide S (LPS), or cellular appendages (Poranen et al., 2002). Subsequently, delivery of the viral genome into the cellular cytoplasm happens through the cell wall and bacterial membrane. Indeed, the nature of the host cell wall has a great influence on the viral entry mechanism and different cell types expose diverse external envelope structures. Three main entry strategies have been reported for viral entry in bacteria: genome release through an icosahedral vertex; dissociation of virion at the cell envelope; and virion penetration via membrane fusion (Poranen et al., 2002). Thus far insights into the mechanisms of entry by archaeal viruses have been based on coincidental observations. However, more recently a few detailed analyses have provided a better understanding of the molecular mechanisms at play in archaeal virus-host systems from geothermal environments.

### Interaction with Cellular Appendages

Filamentous, flexible viruses of the *Lipothrixviridae* family have been classified into four different genera partly based on the virion core and terminal structures. Indeed, the exposed filaments can vary in number from one (AFV9, *Acidianus* filamentous virus 9) to six (SIFV, *Sulfolobus islandicus* filamentous virus) or even form complex structures like claws (AFV1) or brushes (AFV2; Arnold et al., 2000; Bettstetter et al., 2003; Haring et al., 2005b; Bize et al., 2008). The high diversity of terminal structures observed in this particular family strongly suggests their involvement in cellular adsorption processes. Indeed, AFV1 particles terminate with claws that mediate attachment to cellular pili (Bettstetter et al., 2003). In the case of AFV2, the “bottle brush,” a complex collar termini with two sets of filaments, should be able to interact with the surface of host cells directly since its specific host doesn’t show any extracellular appendages (Haring et al., 2005b). In addition, SIFV virions display mop-like structures found in open or closed conformations (Arnold et al., 2000). Hence, lipothrixviruses are decorated with diverse and unique terminal structures that play a major role in recognition and interaction with the host cell.

**FIGURE 1:**
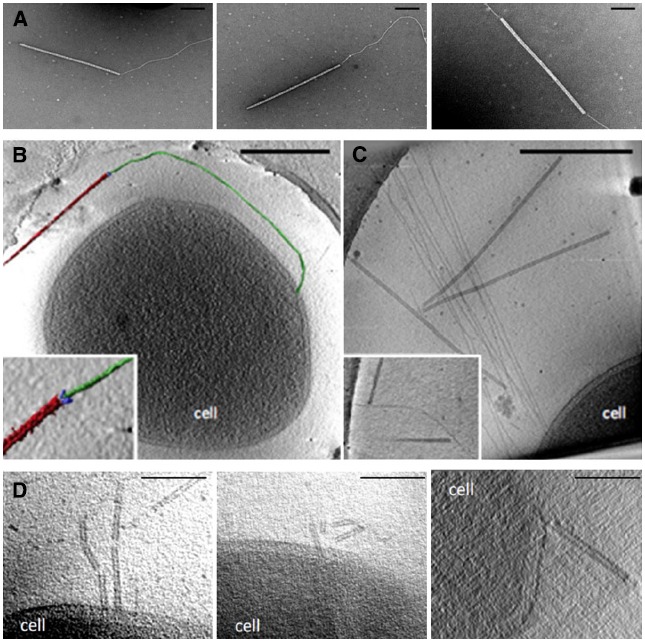
**Entry of SIRV2 in *S. islandicus* LAL14/1 cells. (A)** Transmission electron micrographs showing that SIRV2 virions interact with purified cellular filaments. Stained with 2% uranyl acetate for 2 min. Scale bar, 200 nm. Electron micrographs of SIRV2 interaction with *S. islandicus* LAL14/1 cells. Samples were collected 1 min post-infection and flash-frozen for electron cryotomography (cryo-ET). The virions interact both at the filament tips **(B)** and along the length of the filaments **(C)**. The lower left panel **(B)** also shows a segmented tomographic volume of the SIRV2 virion (red) attached to the tip of an *S. islandicus* filament (green). The three terminal virion fibers that appear to mediate the interaction are shown in blue (the inset depicts a magnified view of the interaction between the virion fibers and the tip of the filament). The inset in the lower right panel **(C)** depicts two virions bound to the sides of a single filament. Scale bars, 500 nm. **(D)** Tomographic slices through *S. islandicus* LAL14/1 cells at 1 min after infection with SIRV2 reveals partially disassembled SIRV2 virions at the cell surface. Adapted from (Quemin et al., 2013). Scale bar, 100 nm.

In a similar manner, the stiff, filamentous rudivirus SIRV2 (*Sulfolobus islandicus* rod-shaped virus 2) was also shown to bind host pili by the three terminal fibers of virions. SIRV2 is one of the more appealing models to study virus-host interactions in archaea (Prangishvili et al., 2013). Recently published analyses concluded that adsorption occurs within the first minute of infection, much more efficiently than in halophilic archaeal systems for which binding requires several hours (Kukkaro and Bamford, 2009). The particles of SIRV2 specifically attach to the tip of host pili-like structures leading to a strong and irreversible interaction between the viral and cellular determinants (Figure 1A). Subsequently, viruses are found on the side of the appendages indicating a progression toward the cell surface where DNA entry is concomitant with virion disassembly (Quemin et al., 2013; Figures 1C,D). Thus, the three fibers located at the virion termini represent the viral anti-receptors involved in recognition of host cells and are responsible for the primary adsorption (Figure 1B). It is noteworthy that both ends of the virions have an equal binding capacity as previously noticed for the lipothrixvirus AFV1 (Bettstetter et al., 2003). The families *Lipothrixviridae* and *Rudiviridae* belong to the order *Ligamenvirales* and are known to attach to extracellular filaments (Prangishvili and Krupovic, 2012). Although AFV1 is capable of binding the side of host pili, a feature shared with bacterial leviviruses, cystoviruses and some tailed bacteriophages (Poranen et al., 2002), the interaction of SIRV2 with *Sulfolobus* filaments occurs initially via the tip. This resembles more closely the primary adsorption observed in the inoviruses (Rakonjac et al., 2011). All these data suggest that linear archaeal viruses employ a common strategy for the initiation of infection although the molecular mechanisms involved are most likely to be distinct.

### Interaction with Cell Surface

As a general rule, viral entry implies direct or indirect binding to the cell surface depending on whether a primary adsorption step is required. In the case of SIRV2, analysis of virus-resistant strains provided interesting candidates for the receptors of SIRV2 virions at the cell surface. In fact, two operons were identified: sso2386-2387 and sso3139-3141 (Deng et al., 2014). The former encodes proteins homologous to components of type IV pili and the latter presumably a membrane-associated cell surface complex. In *S. acidocaldarius*, the assembly ATPase, AapE, and the central membrane protein, AapF, homologous to Sso2386 and Sso2387, respectively, are both essential for the assembly of the type IV adhesive pilus (Henche et al., 2012). The sso3139-3141 operon is thought to encode a membrane bound complex, which could function as a secondary receptor for SIRV2 (Deng et al., 2014).

While entry of rudiviruses, and filamentous archaeal viruses in general, relies on two coordinated adsorption steps, other systems interact spontaneously with the cell surface. As far back as 1984, SSV1 (*Sulfolobus* spindle-shaped virus 1) was reported to exist in different states: isolated particles, incorporated in typical rosette-like aggregates or even bound to cell-derived membrane (Martin et al., 1984). The best known member of the *Fuselloviridae* family displays a lemon-shaped morphotype with terminal fibers at one of the two pointed ends (Stedman et al., 2015). The set of short, thin filaments of the α-fuselloviruses are involved in viral attachment and association with host-derived structures in general. However, the β-fuselloviruses, SSV6 and ASV1 (*Acidianus* spindle-shaped virus 1), exhibit more pleomorphic virions with three or four thick, slightly curved fibers (Krupovic et al., 2014). Although these appendages do not interact with each other as observed for SSV1, some genomic features strongly suggest that the fibers are composed of host-attachment proteins (Redder et al., 2009). Notably, one gene common to all family members (SSV1_C792) and two genes in β-fuselloviruses (SSV6_C213 and SSV6_B1232) encode for the protein responsible for terminal fibers. This protein shares a similar fold with the adsorption protein P2 of bacteriophage PRD1 (Grahn et al., 2002; Redder et al., 2009). In addition, the pointed end of the enveloped virus ABV (*Acidianus* bottle-shaped virus), from the *Ampullaviridae* family, is involved in attachment to membrane vesicles and formation of virion aggregates (Haring et al., 2005a). Therefore, even if data are still scarce, interaction with cellular membranes appears to be a common feature of hyperthermophilic archaeal viruses that contain a lipidic envelope. This particularly interesting feature merits further investigation.

### Release of Viral Genome

Receptor recognition and binding typically induce a cascade of events that start with structural reorganization of the virions and lead to viral genome penetration through the cell envelope (Dimitrov, 2004). Non-enveloped viruses either inject the genome into the cell interior while leaving the empty capsid associated with the cell envelope or deliver the nucleic acids concomitantly with disassembly of the virion at the cell surface. Superficially, the entry of SIRV2 is similar to that of Ff inoviruses or flagellotrophic phages, which bind F-pili and flagella respectively (Guerrero-Ferreira et al., 2011; Rakonjac et al., 2011). First, the interaction with host pili-like structures has been shown and secondly, partially broken particles have been observed at the cellular membrane (Quemin et al., 2013; Figure 1). Notably, no archaeal retraction pili has been identified so far and flagella (called archaella in archaea) of *Sulfolobus* are considerably thicker than the filaments to which SIRV2 binds (Lassak et al., 2012). Additional experiments are needed in order to determine whether the mechanisms of SIRV2 translocation and genome delivery are related to those employed by Ff inoviruses and flagellotrophic bacteriophages, or are completely novel.

Lipid-containing viruses display unusual virion architecture and appear to make direct contact with the plasma membrane. It is reasonable to assume that enveloped viruses rely on a fundamentally different entry mechanism to that employed by non-enveloped filamentous viruses, such as rudiviruses. They might deliver their genetic material into the cell interior by fusion between the cytoplasmic membrane and the viral envelope in a similar fashion to the eukaryotic enveloped viruses (Vaney and Rey, 2011). ATV (*Acidianus* two-tailed virus) resembles fuselloviruses with virions extruded from host cells as lemon-shaped. However, ATV has been classified within the *Bicaudaviridae* partly due to its peculiar life cycle (Haring et al., 2005c). Surprisingly, at temperatures close to that of its natural habitat (85°C), the released tail-less particles show the formation of two long tails protruding from the pointed ends. These extracellular developed tubes contain a thin filament inside and terminate in an anchor-like structure, not observed in the tail-less progeny. The two virion forms, tail-less and two-tailed, were reported to be infectious, thereby indicating that the termini are not involved in the initial stages of infection (Prangishvili et al., 2006b). However, genomic analysis as well as molecular studies highlighted some viral encoded proteins that could be important during infection. For example, the three largest open reading frames (ORFs) and one of the CPs have putative coiled-coil domains, which are usually associated with specific protein–protein interactions and protein complex formation. Moreover, two other proteins carry proline-rich regions (ORF567 and ORF1940) similar to the protein TPX and are abundant during infection by lipothrixvirus TTV1 (*Thermoproteus tenax* virus 1; Neumann and Zillig, 1990). Notably, in particular the motif TPTP has been implicated in host protein recognition for the African swine fever virus (Kay-Jackson, 2004). Finally, pull-down experiments provided evidence for a strong interaction between the ATV protein P529 and OppAss as well as cellular Sso1273, encoding a viral AAA ATPase. The cellular OppAss, an N-linked glycoprotein, is most likely part of the binding components of the ABC transporter system. It is encoded within the same operon and could serve as a receptor. It has also been proposed that the AAA ATPase would trigger ATV host cell receptor recognition. This is based on the hypothetical requirement of its endonuclease activity for the cleavage of the circular viral DNA prior to entry in the cell (Erdmann et al., 2011).

The case of the bottle-shaped virus ABV is also particularly intriguing. The enveloped particles display an elaborate organization with a funnel-shaped body composed of the “stopper,” the nucleoprotein core and the inner core. Presumably, the so-called “stopper” takes part in binding to the cellular receptor and is the only component to which the viral genome is directly attached. Therefore, it has been suggested that the “stopper” could play the role of an “injection needle” in a manner similar to that found in bacterial viruses. Actually, it is well known that head-tail bacteriophages belonging to the *Caudovirales* order use this transmembrane pathway for channeling and delivery of nucleic acids (Poranen et al., 2002). The inner core of ABV virions is the most labile part and could undergo structural changes that would facilitate the release of viral DNA (Haring et al., 2005a). Whether the energy accumulated in the structure after packaging of the supercoiled nucleoprotein is sufficient to transport the whole genetic material into the cytoplasm is unclear. However, relaxation of the nucleoprotein filament, wound up as an inverse cone, concomitantly with its funneling into the cell could be an efficient way of utilizing the energy stored during packaging for DNA injection as previously observed in bacteria (Poranen et al., 2002).

How archaeal viruses interact with the cell surface and deliver the viral genome into the host cytoplasm is still puzzling. Some systems, rudiviruses and lipothrixviruses, show similarities to their bacterial counterparts while others, fuselloviruses, bicaudavirus and ampullavirus, could be related to eukaryotic viruses. Identification of the pathways utilized by both filamentous and unique lipid-containing viruses represents a great challenge and one of the main issues that should be tackled in the near future. It is noteworthy that the S-layer is generally composed of heavily glycosylated proteins and many archaeal viruses exhibit glycosylated capsid proteins. The fact that several glycosyltransferases are encoded in viral genomes (Krupovic et al., 2012) is particularly intriguing. Indeed, protein glycosylation is an important process, which could be involved in virion stability and/or interaction with the host cell (Markine-Goriaynoff et al., 2004; Meyer and Albers, 2013).

## Strategies for Viral Escape from the Host Cell

The last and essential step of the viral infection cycle is escape of viral particles from the host cell. So far, the egress mechanism has been analyzed for only a small subset of archaeal viruses (Torsvik and Dundas, 1974; Bize et al., 2009; Brumfield et al., 2009; Snyder et al., 2013a). Some viruses are completely lytic, while others are apparently stably produced without causing evident cell lysis (Bettstetter et al., 2003). In addition, there are temperate archaeal viruses with a lysogenic life cycle for which induction of virion production in some cases leads to cell disruption (Janekovic et al., 1983; Schleper et al., 1992; Prangishvili et al., 2006b).

The release mechanisms utilized by archaeal viruses can be divided in two categories: those for which the cell membrane is disrupted and those where the membrane integrity remains intact. The strategy for egress is linked with the assembly mechanism of new virions. Some archaeal viruses are known to mature inside the cell cytoplasm and provoke lysis, such as STIV1 (*Sulfolobus* turreted icosahedral virus) and SIRV2 (Bize et al., 2009; Brumfield et al., 2009; Fu et al., 2010). However, most non-lytic viruses undergo final maturation concomitantly with passage through the cell membrane (Roine and Bamford, 2012) or even in the extracellular environment, as observed for ATV (Haring et al., 2005c).

### Cell Membrane Disruption

#### Lysis by Complete Membrane Disruption

Disruption of cell membranes can be caused by lytic or temperate viruses. In case of temperate viruses the cell lysis occurs typically after induction of virus replication and virion formation. Virion production of lysogenic viruses can be induced by various stimuli such as; UV radiation, addition of mitomycin C, starvation or shift from aerobic to anaerobic growth (Janekovic et al., 1983; Schleper et al., 1992; Prangishvili et al., 2006b; Mochizuki et al., 2011).

The first archaeal viruses were isolated from hypersaline environments long before archaea were recognized as a separate domain of life (Torsvik and Dundas, 1974; Wais et al., 1975). These viruses infect halophiles, which belong to the phylum Euryarchaeota. The viral particles exhibit a head-and-tail morphology classical for bacterial viruses. Infection with these viruses resulted in complete lysis of the cells, suggested by a decrease in culture turbidity. Later on, more euryarchaeal viruses were isolated from hypersaline or anaerobic environments, and several of these viruses displayed non-head-tail morphologies such as icosahedral or spindle shapes. Again, in some cases, optical density diminishes with time after viral infection, indicating that a part of these viruses initiate cell lysis (Bath and Dyall-Smith, 1998; Porter et al., 2005; Jaakkola et al., 2012). However, several euryarchaeal viruses apparently do not cause cell lysis.

Amongst hyperthermophilic crenarchaeal viruses there has only been a single report of a decrease in the turbidity of infected cultures (Prangishvili et al., 2006a). In this case, induction of virion production of the lysogenic viruses TTV1-3 led to cell lysis, which was measured by decreasing turbidity (Janekovic et al., 1983). Lysis induced by archaeal viruses can either be coupled with virion production (Jaakkola et al., 2012), or take place after the largest virion burst, therefore raising the possibility of an additional release mechanism in such systems (Bath and Dyall-Smith, 1998; Porter et al., 2005, 2013). Although measurement of optical density is a classical method for the characterization of viral cycles and decrease in turbidity has been observed for several archaeal viruses, no molecular mechanism to achieve complete membrane disruption in archaea has been proposed as yet.

Bacterial virus-host systems are widely studied and as a result the mechanism of lysis used by bacterial viruses is better understood. Bacterial viruses typically induce cell lysis by degradation of the cell wall, which is achieved by muralytic endolysins (Young, 2013). In addition, most bacterial viruses encode small proteins named holins (Bernhardt et al., 2001a,b; Catalao et al., 2013). Holins usually accumulate harmlessly in the bacterial cell membrane until a critical concentration is reached and nucleation occurs. Nucleation results in formation of two dimensional aggregates, “holin rafts,” that rapidly expand and create pores in lipid layers through which the endolysins can reach the cell wall (Young, 2013). In gram negative bacteria the presence of an outer membrane requires additional virus-encoded proteins, spanins, which are suggested to induce fusion of the inner and outer membrane (Berry et al., 2012). After an initial degradation of the peptidoglycan cell wall, the cells burst due to osmotic pressure, explaining total loss of turbidity observed for infected bacterial cultures (Berry et al., 2012). Accurate timing of lysis is essential for successful virus reproduction and is achieved by regulation of holin expression (Young, 2013). Since archaea lack a peptidoglycan cell wall, endolysin-holin egress systems are not effective in archaea. Only a few archaeal species contain a peptidoglycan-like cell wall consisting of pseudomurein polymers (Albers and Meyer, 2011). The oligosaccharide backbone and amino acid interbridges of murein and pseudomurein are different, rendering bacterial endolysins ineffective to pseudomurein (Visweswaran et al., 2011). However, pseudomurein degrading enzymes are encoded by a few archaeal viruses infecting methanogens; the integrated provirus ψM100 from *Methanothermobacter wolfeii* and the virus ψM1 infecting *M. marburgensis* (Luo et al., 2001). How these intracellularly produced viral endolysis traverse the archaeal cell membrane in order to degrade the pseudomurein cell wall is not clear, since the mandatory pore forming holins have not been identified in the genomes of these viruses. The possible presence of archaeal holins could be currently overlooked, as genes encoding holins share generally very little sequence similarity, making it difficult to predict their presence in genomes (Saier and Reddy, 2015).

The large majority of archaea lack a pseudomurein cell wall. Therefore instead of a endolysin-holin system, a fundamentally different lysis mechanism would be required for release of virions from these cell wall lacking archaea. One hypothesis is that archaeal viruses employ holins to disrupt the cell membrane, possibly combined with proteolytic enzymes in order to degrade the S-layer. To date there are about a dozen holin homologs identified in archaeal genomes based on sequence similarity (Reddy and Saier, 2013), but none of the predicted proteins have been tested *in vivo*. Moreover, not a single holin-encoding gene has been identified in the genomes of currently isolated archaeal viruses (Reddy and Saier, 2013; Saier and Reddy, 2015). In addition, specific enzymes capable of S-layer degradation are currently unknown and S-layer proteins and sugars display a large diversity in different species (Albers and Meyer, 2011). Thus in contrast to bacterial endolysins that degrade peptidoglycan cell walls of virtually all bacteria, specific tailor made proteases would be required to degrade archaeal S-layers of different species.

#### Lysis by Formation of Defined Apertures

The egress mechanism of only two archaeal viruses (STIV1 and SIRV2) has been studied in high molecular detail. Both employ a release mechanism that relies on the formation of pyramidal shaped egress structures, which are unique to archaeal systems (Bize et al., 2009; Brumfield et al., 2009; Quax et al., 2011; Snyder et al., 2011). At first glance, both viruses were regarded as non-lytic viruses, since a decrease in cell culture turbidity was never observed (Prangishvili et al., 1999; Rice et al., 2004). However, the use of several electron microscopy techniques clearly showed that the two viruses induced cell lysis (Bize et al., 2009; Brumfield et al., 2009). Their particular lysis mechanism yields empty cell ghosts explaining the maintenance of culture turbidity.

**FIGURE 2:**
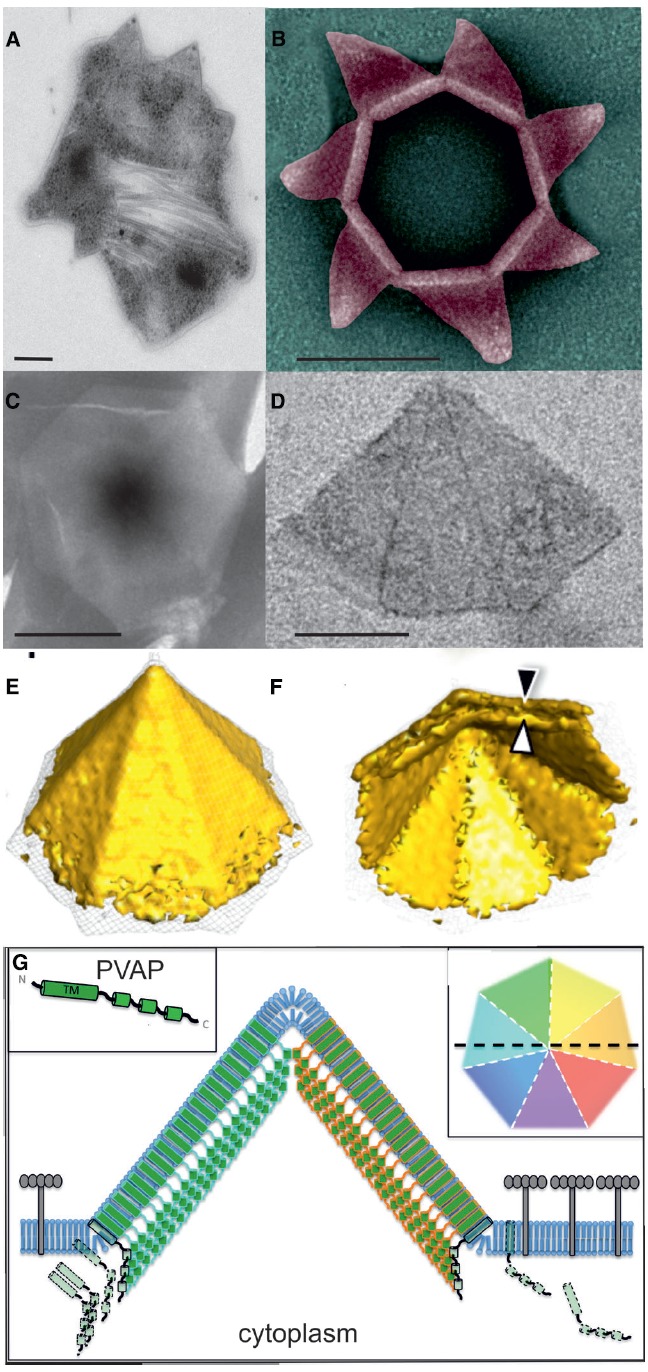
**Remarkable archaeal virion egress structure. (A)** Scanning electron micrograph of an SIRV2 infected *S. islandicus* cell displaying several VAPs. **(B)** Transmission electron micrographs of isolated VAPs in closed and **(C)** open conformation. **(D)** Solid representation of VAP obtained by subtomogram averaging displaying the **(E)** outside and **(F)** interior. **(G)** Model of VAP formation. Adapted from (Bize et al., 2009; Quax et al., 2011; Daum et al., 2014). Scale bar, 100 nm.

Infection by SIRV2 and STIV1 leads to formation of several pyramidal shaped structures on the cell membrane of *S. islandicus* and *S. solfataricus* respectively (Bize et al., 2009; Brumfield et al., 2009; Prangishvili and Quax, 2011; Figure 2A). These virus-associated pyramids (VAPs) exhibit sevenfold rotational symmetry and protrude trough the S-layer (Quax et al., 2011; Snyder et al., 2011; Figures 2B–D). At the end of the infection cycle, the seven facets of the VAPs open outward, generating large apertures through which assembled virions exit from the cell (Fu et al., 2010; Quax et al., 2011; Daum et al., 2014; Figure 2B). The baseless VAP consist of multiple copies of a 10 kDa viral encoded protein, PVAP (STIV1_C92/SIRV2_P98) (Quax et al., 2010; Snyder et al., 2013a). This protein contains a transmembrane domain, but lacks a signal sequence and seems to be inserting in membranes based on hydrophobicity of its transmembrane domain (Quax et al., 2010; Daum et al., 2014). PVAP has the remarkable property to form pyramidal structures in virtually all biological membranes, as was demonstrated by heterologos expression of PVAP in archaea, bacteria and eukaryotes (Quax et al., 2011; Snyder et al., 2013a; Daum et al., 2014).

Nucleation of the PVAP-induced structure starts on the cell membrane, most likely with the formation of a heptamer of PVAP subunits (Daum et al., 2014). The structures develop by the outward expansion of their seven triangular facets. They reach sizes of up to 200 nm in diameter, both in natural and heterologous systems (Quax et al., 2011; Daum et al., 2014). In contrast to bacterial holin rafts, the formation of VAPs is not a sudden process depending on a critical protein concentration. PVAP transcripts steadily increase throughout the infection cycle and PVAP integrates in the membrane until late stages of infection (Quax et al., 2010, 2013; Maaty et al., 2012). Although VAPs are slowly formed, their actual opening is quite rapid (Bize et al., 2009; Brumfield et al., 2009; Snyder et al., 2011; Daum et al., 2014). The nature of the signal triggering this opening has not been identified yet. VAPs, formed after heterologous PVAP expression, in bacteria and eukaryotes were never observed in open conformation, suggesting that an archaeal specific factor is required (Daum et al., 2014). It has been proposed that the archaeal ESCRT (Endosomal Sorting Complex Required for Transport) machinery could be involved in the STIV1 VAP-based exit (Snyder et al., 2013b). Considering that genes encoding ESCRT machinery are specifically down regulated during SIRV2 infection (Quax et al., 2013), and that STIV1 contains in contrast to SIRV2 an inner lipid layer (Veesler et al., 2013), STIV1 requirement of the ESCRT system might be independent from VAP-induced lysis.

The ultrastructure of VAPs of SIRV2 was studied by whole cell cryo-tomography and subtomogram averaging. This revealed the presence of two layers, of which the outer one is continuous with the cell membrane and presumably formed by the N-terminal transmembrane domain (Daum et al., 2014; Figures 2E,F). The inner layer represents a protein sheet formed by tight protein–protein interactions of the C-terminal domain of the protein (Daum et al., 2014). The strong interactions between PVAP monomers are suggested to exclude most lipids and membrane proteins from the VAP assembly site, in a similar fashion as holin raft formation (White et al., 2011; Figure 2G). S-layer proteins are anchored in the membrane, and consequently will be excluded from the VAP assembly site, providing a strategy for VAP protrusion through the S-layer.

The described VAP-based egress mechanism is archaeal specific. Homologues of PVAP are only found amongst some archaeal viruses (Quax et al., 2010). However, the majority of archaeal viruses lack PVAP, suggesting that they rely on a different and as yet unknown mechanism for egress.

### Viral Extrusion without Membrane Disruption

While the first isolated archaeal viruses were lytic, subsequent characterization of more viruses revealed that the large majority do not cause lysis of the host cell. To date, lytic viruses make up half of the viruses infecting euryarchaeota, and only three in crenarchaea (Torsvik and Dundas, 1974; Wais et al., 1975; Janekovic et al., 1983; Bize et al., 2009; Brumfield et al., 2009; Pina et al., 2011). In addition, some studies indicate that free virions can be observed before disruption of archaeal cells, suggesting that another egress mechanism exists, which preserves cell membrane integrity. It might be possible that some lytic archaeal viruses have been currently overlooked due to special characteristics of their lysis mechanism, as was the case for STIV1 and SIRV2 (Prangishvili et al., 1999; Rice et al., 2004). Nevertheless, the low number of lytic archaeal viruses contrasts with the situation in bacteria, for which lytic viruses are very common. The majority of archaeal viruses are thought to be continuously produced without integrating into the host genome or killing their hosts (Pina et al., 2014). This equilibrium between viruses and cells is referred to as a “stable carrier state” (Bettstetter et al., 2003; Prangishvili and Garrett, 2005; Prangishvili et al., 2006a). The nature of this stable carrier state and the mechanisms by which virions are extruded from archaea without causing cell lysis, remain poorly understood.

In contrast to the situation in archaea, the majority of bacterial viruses are lytic. Almost all bacterial viruses exit via the holin based mechanism described above. However, an exception to the rule are the bacterial filamentous viruses belonging to the *Inoviridae* that egress without causing cell lysis (Rakonjac et al., 2011). The majority of the inoviruses infect gram negative bacteria. Assembly of inoviruses is finalized during particle extrusion. The interaction between the packaging signal of the viral genome and the cellular membrane initiates the exit step (Russel and Model, 1989). Virally encoded proteins are thought to form pores in the inner membrane through which the DNA is extruded. Multiple copies of the major CP accumulate in the inner membrane and associate with the ssDNA viral genome while it is passing through the virus-induced pores (Rakonjac et al., 1999). A barrel-like structure in the outer membrane permits the release of progeny and is composed of multiple copies of a virus-encoded protein with homology to proteins of type II secretion systems and type IV pili (Marciano et al., 2001). Alternatively, other inoviruses use the host secretion machinery to traverse the outer membrane (Davis et al., 2000; Bille et al., 2005). Even though replication of the viral genome and constituents might burden the cell, the infection of inoviruses does not lead to cell death and is a continuous process. There are several archaeal filamentous viruses known. However, filamentous archaeal viruses are not related to the bacterial inoviruses, nor encode homologs of the secretion-like proteins involved in egress of inoviruses (Janekovic et al., 1983; Bize et al., 2009; Quax et al., 2010; Pina et al., 2014). Therefore the filamentous archaeal viruses must rely on an alternative mechanism for viral extrusion from the cell.

Interestingly, lipid-containing archaeal viruses are quite common (Roine and Bamford, 2012). There are some archaeal icosahedral viruses that possess an inner membrane, such as STIV and SH1 (Bamford et al., 2005; Khayat et al., 2005; Porter et al., 2005). In addition, the filamentous lipothrixviruses (Janekovic et al., 1983; Arnold et al., 2000; Bettstetter et al., 2003), the spherical virus PSV (*Pyrobaculum* spherical virus; Haring et al., 2004) and the pleiomorphic euryarchaeal viruses (Pietila et al., 2009, 2013) all contain an external lipid envelope. The lipids are typically derived from the host cell. Several eukaryotic viruses contain a membrane that is usually obtained during “budding,” a process by which particles egress without disturbing the membrane integrity. Eukaryotic enveloped viruses either encode their own scission proteins, or hijack vesicle formation machinery of their host (Rossman and Lamb, 2013). Archaea are also reported to produce vesicles (Soler et al., 2008; Ellen et al., 2011), and the machinery responsible for vesicle production might be utilized by lipid envelope containing viruses in archaea as well. In particular, the pleiomorphic viruses infecting euryarchaea are likely to be released through budding as their envelope has the same lipid composition as the host they infect (Pietila et al., 2009; Roine et al., 2010).

The most common scission machinery employed by eukaryotic viruses is the ESCRT system (Votteler and Sundquist, 2013). In eukaryotes these proteins are responsible for endosomal sorting in the multi vesicular body. Well-characterized viruses such as Ebola and human immunodeficiency virus (HIV) use the ESCRT proteins during egress (Harty et al., 2000; Weissenhorn et al., 2013). Interestingly, proteins homologous to ESCRT components have been identified in several archaea, where they are involved in cell division (Lindas et al., 2008; Samson et al., 2008; Makarova et al., 2010; Pelve et al., 2011). These proteins represent potential players in budding-like extrusion processes in archaea. The mechanism underlying the release of temperate archaeal viruses remains largely unexplored and represents an appealing area of research that should shed light on original and unconventional strategies.

## Concluding Remarks

The last few years have shown a steady increase in an understanding of archaeal virus-host interactions, therefore revealing the first insights into viral interactions with the archaeal membrane. Viruses have developed various strategies to cross the membrane. These strategies are adapted to the nature of the cell envelope of their host. Some archaeal viruses employ fascinating novel mechanisms, while others appear to rely on processes that at first sight are analogous to their bacterial counterparts. Additional research will help to determine to which extent bacterial, eukaryotic and archaeal virospheres are evolutionary related. The uniqueness of the archaeal cell surface, and the diversity of the currently described archaeal entry and egress mechanisms, argue in favor of future discovery of more innovative and surprising molecular mechanisms.

### Conflict of Interest Statement

The authors declare that the research was conducted in the absence of any commercial or financial relationships that could be construed as a potential conflict of interest.
